# Systems Biology: The Next Frontier for Bioinformatics

**DOI:** 10.1155/2010/268925

**Published:** 2011-02-09

**Authors:** Vladimir A. Likić, Malcolm J. McConville, Trevor Lithgow, Antony Bacic

**Affiliations:** ^1^Bio21 Molecular Science and Biotechnology Institute, The University of Melbourne, Parkville, VIC, 3010, Australia; ^2^Department of Biochemistry and Molecular Biology, The University of Melbourne, Parkville, VIC, 3010, Australia; ^3^Department of Biochemistry and Molecular Biology, Monash University, Clayton, VIC, 3800, Australia; ^4^Australian Centre for Plant Functional Genomics, School of Botany, The University of Melbourne, Parkville, VIC, 3010, Australia

## Abstract

Biochemical systems biology augments more traditional disciplines, such as genomics, biochemistry and molecular biology, by championing (i) mathematical and computational modeling; (ii) the application of traditional engineering practices in the analysis of biochemical systems; and in the past decade increasingly (iii) the use of near-comprehensive data sets derived from ‘omics platform technologies, in particular “downstream” technologies relative to genome sequencing, including transcriptomics, proteomics and metabolomics. The future progress in understanding biological principles will increasingly depend on the development of temporal and spatial analytical techniques that will provide high-resolution data for systems analyses. To date, particularly successful were strategies involving (a) quantitative measurements of cellular components at the mRNA, protein and metabolite levels, as well as *in vivo* metabolic reaction rates, (b) development of mathematical models that integrate biochemical knowledge with the information generated by high-throughput experiments, and (c) applications to microbial organisms. The inevitable role bioinformatics plays in modern systems biology puts mathematical and computational sciences as an equal partner to analytical and experimental biology. Furthermore, mathematical and computational models are expected to become increasingly prevalent representations of our knowledge about specific biochemical systems.

## 1. Introduction

The term “systems biology” has emerged recently to describe the frontier of cross-disciplinary research in biology [1–5]. This term was propelled into the mainstream merely ten years ago [1–3], coinciding with the completion of the Human Genome Project (HGP) [6, 7] and the concomitant emergence of ‘omics technologies, namely transcriptomics [8, 9], proteomics [10], and metabolomics [11, 12]. However, the origins of modern systems biology can be traced back to the middle of last century [13–15], with history that is both conceptually complex and institutionally convoluted. For example, a general systems theory was developed and applied to biology in late 1960's [14, 15]. Independently, the theory of metabolic control was developed, and metabolic flux was recognized as a “systemic property” [16–18]. Here, we focus on the reemergence of “systems thinking” linked to the post-genomic era and the development of global molecular profiling methods collectively known as ‘omics technologies. The discussion of systems biology in the broader historical context can be found elsewhere (see, e.g., [4, 19] and references therein).

**Figure 1:**
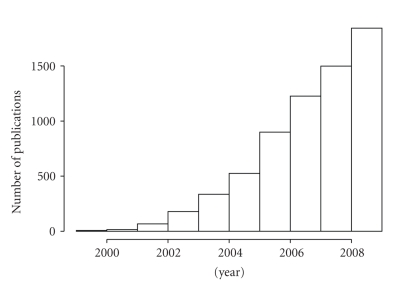
The number of publications referencing “systems biology” in the PubMed database by year (2000–2009). In 2009, over 1,500 such publications appeared in PubMed.

Interest in systems biology has increased rapidly in the past decade, as evidenced by the number of referencing publications (Figure 1). Systems biology has fuzzy boundaries and overlaps with several emerging, post-genomic fields, such as synthetic biology [20–24], systems microbiology [25], systems biotechnology [26, 27], integrative biology [26, 28], systems biomedicine [29], and metagenomics [25, 30]. Numerous definitions of systems biology have been proposed [1, 2, 4, 5, 31], but to date, there is no universally accepted definition—reflecting the difficulty in defining a heterogeneous school of thought by a comprehensive yet concise definition. Each of the proposed definitions, however, revolves around a fundamental understanding of biological systems based on the underlying component interactions (molecular interactions, in the case of biochemical systems biology). In a broad sense, the same is the goal of more traditional disciplines, such as molecular biology, genomics, and biochemistry. Hence, the question “*what is new in Systems Biology?*” has been extensively discussed (see, e.g., [1–5, 31, 32]). Furthermore, it has been argued that systems biology is an approach, rather than a scientific discipline in the traditional sense [31–33]. While the room for future debate on these questions remains, it is clear that systems biology fundamentally depends on the applications of mathematical and computational modeling. As the computational applications in biology are most often associated with the province of bioinformatics, another relevant question is: “what is the relationship between systems biology and bioinformatics?”. Here, we address this question by focusing on the recent, post-HGP history, and the reemergence of biochemical systems biology.

## 2. From Genomics to Systems Biology

The term “genomics” was coined by Thomas Roderick in 1986, and soon after was adopted as the name of the new journal aimed to support the new discipline of genome mapping and sequencing [34]. This was a time of great excitement and profound transformation in biology brought about by the development of increasingly efficient methods for DNA sequencing [35–37]. At the time, the call for the sequencing of the human genome was gaining momentum [38, 39], and in 1988, the National Research Council of the US Academy of Sciences recommended the initiation of the Human Genome Project [39]. The HGP, completed a decade later, was an enormous success, thus validating the new discipline of genomics. It rallied the scientific community in unprecedented ways, from being a global collaboration of 20 sequencing centers from six countries to opening new horizons in large-scale biology [39]. The momentum of the HGP has spurred a plethora of genome-sequencing projects of other organisms, including plants, animals, and microorganisms. In the early phases, the sequencing projects focused mainly on mapping, sequencing, and identifying genes [40]. As the various genome-sequencing projects gathered momentum, it has become clear that collected genome sequences were only revealing more of hidden complexity, and are opening new and deeper biological questions [40, 41]. As a result, an increasing emphasis was placed upon the relationship between the sequence and function, and the field of genomics started to differentiate into “sequence genomics” and “functional genomics” [40, 42, 43].

The early view underpinning genomics was that the genome, the ultimate sequence map of the organism's DNA, is “a rosetta stone from which the complexities of gene expression in development can be translated and the genetic mechanisms of disease interpreted” [34]. This simplistic view rested on the deterministic concept of a gene and its role in determining biological function and organism's phenotype, the notion of which was tacitly extended to the entire genome. The degree of elusiveness of the gene concept has become fully apparent only in the last decade [44–48], based on the analysis of sequenced genomes, and extensive studies of the transcriptome with new techniques (such is cap-analysis gene expression (CAGE) and tiling arrays [49, 50]). Several facts highlight the complexity of the relationship between the organism's phenotype and its genome: (i) less than 2% of human DNA directly encodes proteins [51], (ii) the genomes of eukaryotic organisms are nearly entirely transcribed [50, 52], (iii) a massive amount of noncoding RNA transcripts identified in higher organisms is thought to have an important regulatory role [53]; and (iv) a critical importance of post-transcriptional and post-translational regulation in the control of the function of gene products, which is both spatially and temporally regulated [54]. As a result, in the past five years, the concept of the gene has been subject of substantial revisions [44–48].

Only temporarily overshadowed with the excitement about generating genome sequences, the true complexity of the relationship between an organism's genome and phenotype was recognized early. Even in the initial development of genomics we can recognize the elements of “systems” thinking. In 1997, Hieter and Boguski wrote “*Functional genomics will … supplement the detailed understanding of gene function provided by traditional approaches with a powerful new perspective on the holistic operation of biological system*s” [40]. In the next few years, the idea of a “holistic understanding” was further articulated in terms of mathematical models, whole-genome data sets, and the experience accumulated in studies of complex systems [1–3]. Almost simultaneously, the need for an engineering mindset in molecular biology was suggested in an influential (and humorous) article written by a prominent biologist [55]. An aspect of systems thinking is the recognition that biological systems are “complex” in the mathematical sense [56–58]. Such complex systems have long been of interest in physics and mathematics, and the direct relevance of the knowledge accumulated in these disciplines to biology was realized [58, 59]. It is now widely recognized that the availability of fully sequenced genomes and high-throughput (‘omics) data sets makes the aspirations of “systems” thinking in biology an achievable goal [60–64].

## 3. System-Level Description, System-Level Understanding, and the System Itself

There are two frequently quoted approaches to systems biology, namely, “top-down” and “bottom-up” [5, 65, 66]. Furthermore, systems biology practitioners can be arbitrarily divided into two (not mutually exclusive) camps: “pragmatic” and “systems oriented” [67, 68]. O'Malley and Dupre suggested that both camps of systems biologists lack a clear definition of what constitutes a “system” [68]. Indeed, the literature abounds with different definitions and calls for “system-level description” and “system-level understanding”. This only confounds the matter since the universally accepted definition of “system” is lacking. While confusing at first sight, the meaning of “system” in systems biology depends on the problem at hand, the objectives of the study, and the choices made in the art of mathematical modeling.

Mathematical modeling is often used in genomics and molecular biology, but in systems biology, it takes center stage, as “no more, but no less, than a way of thinking clearly” [69]. Biological systems consist of a large number of functionally diverse components, which interact highly selectively and often nonlinearly to produce coherent behaviors [2]. These components may be individual molecules (such as in signaling or metabolic networks), assemblies of interacting complexes, sets of physical factors that guide the development of an organism (genes, mRNA, associated proteins and protein complexes), cells in tissues or organs, and even entire organisms in ecological communities. What is common to all these examples is the sheer number of components, and their selective, non-linear interactions that render the behaviors of these systems beyond the intuitive grasp. Take, for example, the cell cycle in the yeast *Schizosaccharomyces pombe*: the model of its cell-cycle regulatory network involves about twenty components, whose interactions can be *approximately* described with a dozen differential equations and about 30 kinetic parameters [70]. The dynamic behavior of this network of interactions is possible to grasp only with the help of computer simulations and dynamical systems theory [70, 71]. Another example is the cellular response of yeast to hyperosmotic shock: it is only with mathematical modeling that a coherent picture emerges, connecting various known components of the system with the observed properties [72].

There are other reasons why the concept of “system” is so elusive. The role of mathematical models, particularly in generating experimentally testable hypotheses, has been discussed extensively [2, 5, 19]. Perhaps less widely appreciated is that mathematical models of biological systems are increasingly being used to represent our knowledge about these systems. For example, the *i*AF1260 model of *Escherichia coli*'s metabolic network not only predicts experimentally observed behavior of *E. coli* under genetic perturbations [73], but also in itself is a representation of the *E. coli* metabolic network. Similarly, the kinetic model of glycolysis in the bloodstream form of *Trypanosoma brucei* [74] is the state-of-the-art representation of glycolysis in this organism. There is no alternative way of quantitative thinking about these complex systems but through models that rely on precise mathematical descriptions. These mathematical or computational models are essentially beyond a simple intuitive grasp, and represent concise summaries of our current knowledge of respective systems.

There may be significant differences in scope and scale between different models used in systems biology. Consider, for example, the model of the yeast genome-scale metabolic network [75] and the model of glycolysis in yeast [76]. It is not that one model is better than the other, rather the two models have different motivations, objectives, scales, and capabilities: the first is the genome-scale model of the entire metabolic network, while the second is a model of a single metabolic pathway which includes detailed descriptions of kinetics of individual enzyme catalyzed reactions. This illustrates an important general principle of mathematical modeling, highly relevant to systems biology: every mathematical model aims for a certain level of description, which depends on the objective of the study, limitations in our knowledge about the system of interest, and our ability to experimentally observe the system/phenomena of interest (necessary for testing the model's predictions).

Genome-scale metabolic models typically ignore kinetic parameters of individual reactions because such models aim to be comprehensive, and the kinetic parameters for most reactions are unknown (but see recent theoretical advances [77]). In contrast, kinetic models are much more detailed but less comprehensive; however, they can provide not only the information about the steady state but also the time course given some initial conditions. Choosing the correct “level of description” is one of the more difficult aspects in mathematical modeling and is a pervasive challenge in systems biology. Another challenge is choosing the boundaries of the model (note: this amounts to defining the “system”). This usually requires exquisite familiarity with the phenomena of interest, and a considerable experience in mathematical modeling. Complex dynamical systems form structures [59], and nature often provides modular designs [78]. This modularity must be both understood and exploited correctly for optimal modeling. In genome-scale studies of microbial organisms, a convenient system boundary is the cell boundary; in most other cases, the question of the appropriate systems boundary is more opaque and must be addressed based on the prior knowledge of components and the coupling between these components. Trivial examples of this include tissue structure in a multicellular organism or subcellular compartmentalization of metabolites. Hence all the difficulties in defining the “system” in systems biology.

Modern systems biology is a rapidly evolving discipline. In the past, systems thinking was invoked in the context of a variety of systems and processes: from humans [79, 80] to microorganisms [5, 60, 81], animals [82, 83], and plants [84], and in regards to different levels of biological organization, from molecular subnetworks [70, 74, 76, 85, 86], to cellular interaction networks [87], cells, entire organs [88], organisms [80, 83], and even communities of organisms [25, 30]. Areas that have proven particularly fruitful for systems biology include studies of biochemical networks and applications to microorganisms [60, 61, 73, 89–93].

## 4. Biochemical Networks in Microorganisms

**Figure 2:**
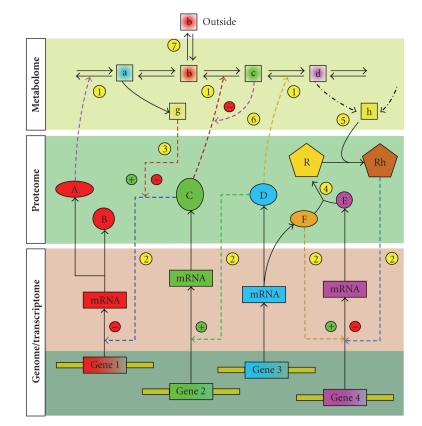
A conceptualization of biochemical networks showing genome, transcriptome, proteome, and metabolome-level networks, highlighting their complexity and mutual interdependence. In biological systems a large number of structurally and functionally diverse components (genes, proteins, metabolites) are involved in dynamic, non-linear interactions, which in turn involve a range of time scales and interaction strengths. Direct conversions of species shown in solid lines, while some possible interactions (not necessarily one-step) are designated in dashed lines. Several types of interactions are shown: (1) enzyme catalysis, (2) posttranscriptional control of gene expression by proteins/protein complexes, including mechanisms that act on mRNAs (deadenylation, storage granulation) and mechanisms that act either directly or indirectly on DNA (histone modification, methylation), (3) effect of metabolite on gene transcription mediated by a protein, (4) protein-protein interaction, (5) effect of a downstream (“reporter”) metabolite on transcription through binding to a protein, (6) feedback inhibition/activation of an enzyme by a downstream metabolite, and (7) exchange of a metabolite with outside of the system (cell, organism).

We are only beginning to appreciate the full complexity and the multidimensional nature of biochemical networks operating in all living organisms (Figure 2). Studies of metabolic networks, gene regulatory networks, and protein-protein interaction networks in microbial organisms have significantly contributed to this, and indeed to the identity of systems biology. Microorganisms are convenient models for systems studies for several reasons: (i) decades of genetic and biochemical work have generated deep biological insights, and resulted in sophisticated molecular biology techniques for experimental manipulation, (ii) they can be readily and rapidly cultured in inexpensive media, providing ample material for controlled experiments, (iii) many are pathogens of humans, plants, and domestic animals, and therefore are of medical or environmental interest, and (iv) many are important in industrial processes and therefore are relevant for biotechnology. Furthermore, microbial organisms are unicellular, and the cell membrane provides a convenient boundary that delineates the “system” for genome-wide studies. In the past, microbes have been used in numerous systems studies, including that of genetic networks [90, 94], protein-protein interactions [89, 95, 96], metabolic networks [97–100], cell cycle regulation [70, 71], and signal transduction networks [101]. 

Three types of biochemical networks, roughly corresponding to three different levels or “omes”, have been mostly studied in the past: gene regulatory networks [90, 94, 102], protein interaction networks [89, 95, 96, 103] and metabolic networks [97–100, 104]. When prior knowledge of modularity allowed the assumption of decoupling, systems studies on biochemical subnetworks or cross-networks were possible. Examples of this include modeling of the cell cycle in yeast [70, 71], specific metabolic pathways [74, 76, 85], and signal transduction pathways [72, 101, 105]. Several pioneering studies integrated responses across individual “omes”. Examples of this include studies of transcriptome and proteome responses to perturbations in metabolic pathways [61, 106], the effects of a transcriptional regulator on central carbon metabolism in *Bacillus subtilis* [107], and coordinated analysis of the minimal bacterium *Mycoplasma pneumoniae*, including analysis of its mRNA [91], protein complexes [92], and the metabolic network [93]. 

What are these studies telling us? Integrating the information from different biological levels reveals complex and unanticipated global behaviors in what were thought to be “simple” organisms and biochemical systems. For example, the metabolic network in *E. coli* appears remarkably stable with respect to various types of perturbations, but the mechanism for how this stability is achieved appears profoundly different for environmental and genetic perturbations [106]. Surprisingly, the flux through the *E. coli* pentose phosphate pathway is reversed in response to a blocking mutation, and yet, this is achieved with only subtle changes in the enzyme levels [106]. Another telling example is the smallest self-replicating organism, the bacterium *M. pneumoniae* whose genome encodes merely 689 proteins [93]. Compared to more complex bacteria (*E. coli* encodes ~4,200 proteins), *M. pneumoniae* lacks most transcription factors and other regulators; yet this organism shows a highly complex, intrinsically structured transcriptional response, with many alternative transcripts and multiple regulators per gene [91]. In spite of its minimal genome, the proteome of *M. pneumoniae* exhibits modularity and extensive reuse of functional components, with a substantial crosstalk between different cellular processes [92]. Furthermore, *M. pneumoniae* shows highly coordinated changes in gene expression, specific responses to metabolic perturbations, and adaptability to carbon sources similar to that observed in *E. coli* [93]. It is unlikely that *M. pneumoniae* is a fundamentally unusual organism; rather, these observations suggest a host of unknown regulatory mechanisms that operate across the levels of transcriptome, proteome, and metabolome [108].

As a result of decades of detailed biochemical work, metabolic networks are the best understood of all biochemical networks [109, 110]. We have near-complete collections of components and topologies of metabolic networks in model microorganisms such as *E. coli* [73] and *Saccharomyces cerevisiae* [75]. For the model organism *E. coli*, the majority of metabolic reactions, enzymes, cofactors, substrates, and products are known [73]. This, however, represents only the first step towards understanding how these components function in spatial and temporal integration, and precisely what are the controls exerted on them. While the topologies of metabolic networks are well understood, we are only beginning to understand interactions that control metabolism [111, 112]. Metabolite equilibrium concentrations are accessible experimentally through quantitative metabolomic approaches [11, 12, 113], which is directly comparable to the measurement of mRNA and protein levels in transcriptomics and proteomics, respectively. In contrast to all other types of biochemical networks, experimental approaches for assessing *in vivo* reaction rates (fluxes) are also well developed for metabolic networks [109, 114–116]. This is of great importance, as metabolic fluxes are the key determinants of cellular physiology and cannot be predicted from mRNA, protein, or even metabolite levels [114]. Thus, measurement of metabolic flux is equivalent to the measurement of information flow through a signaling pathway, or the information flow between genes residing on the same control circuit. New theoretical frameworks for more efficient extraction of information from experimental data continue to be proposed [117], and a considerable progress has been made in the analysis of metabolic fluxes under isotopic nonstationary conditions [115, 118]. Since nonstationary flux analysis relies on shorter, transient experiments, this opens an array of new possibilities for flux analysis in higher organisms, improving the scope of systems biology studies of metabolic networks [118].

## 5. Bioinformatic Tools for Systems Biology

While many systems biology approaches involve mathematical and computational modeling, the development, maintenance, and dissemination of tools for systems biology is in itself a significant challenge. Examples of this include development of data repositories, data standards and software tools for simulation, analysis and visualization of system components such as biochemical networks. Another example are applications of high-throughput molecular profiling technologies which often require sophisticated data processing and analysis, and typically involve elements of signal processing and statistical analysis. As the resulting quantitative measurements are transferred to formal mathematical models for the purpose of modeling, the endeavor becomes perhaps more systems biology and less bioinformatics. However, that is only a matter of a degree, with often no clear boundary between bioinformatics and systems biology.

The need for effective exchange of formal, quantitative systems biology models has driven the development of the Systems Biology Markup Language (SBML) [119]. The SBML project aims for the development of the computer-readable format for the representation of biological processes. SBML provides a well-defined format which different software tools can use for the exchange of biological models with high fidelity. A testimony to the importance of SBML is its adoption by software tools concerned with biological modeling (at the time of this writing, over 180 software tools support SBML). The graphical notation for the representation of biological processes has been proposed recently (Systems Biology Graphical Notation, SBGN) [120]. The current SBGN specification consists of three complementary languages which aim to describe biological processes and relationships between biological entities [120].

Since studies of biochemical networks are particularly successful aspect of systems biology, it is not surprising that a plethora of computational tools that address different needs in the analysis of biochemical networks have been reported, and in many cases, these tools are freely accessible. Without attempting to be comprehensive, we highlight some of the widely used research and training tools. Systems Biology Workbench (SWB) is a framework that allows different components for systems biology to communicate, exchange models via SBML, and reuse capabilities without understanding all the details of the each component implementation [121]. From the user's perspective, SWB is a collection of tools for systems biology that includes programs for building, viewing, and editing of biochemical networks, tools for simulation, and tools for import and translation of models. Another highly useful tool is CellDesigner, a Java-based program for constructing and editing of biochemical networks [122]. Recent versions of CellDesigner are able to import models in SBML and support display of biochemical networks based on process diagram language specified by SBGN. In CellDesigner models can be simulated either with a built-in simulator, or alternatively CellDesigner can connect to external simulators, such as those provided by SWB [121]. An independent simulator of models encoded in SBML is COPASI [123]. COPASI can simulate models based on ordinary differential equations (ODEs) as well as stochastic models by using the Gillespie's algorithm. COPASI provides tools for visual analysis of simulation results, and can also perform steady-state and metabolic control analyses [123].

As biological research accelerates through the development of new technologies and instrumentation, biological databases have become an indispensable partner in such research. Building and maintaining of primary databases such as GenBank [124] or Protein Data Bank [125] have long been recognized as important bioinformatics work. Primary biological databases serve both as repositories of experimentally derived information and are the basis for the development of secondary databases that capture higher-level knowledge. An example of such secondary database is Pfam database of proteins families and domains [126]. Concomitantly with the development of the biochemical systems biology, an important niche of secondary biological databases has emerged: the databases that capture the properties and processes in biochemical networks. The ecosystem of such databases and associated tools is rapidly growing and includes metabolic pathways databases organized around the BioCyc project [127], database of human biological pathways [128], database of interactions between small molecules and proteins [129], and databases of protein-protein interactions [130]. As these databases attempt to reconstruct and organize information about interactions between cellular components, they also attempt to build higher-level knowledge and theories about the biological processes they are concerned with. Such *in silico* knowledge is much needed, as the integral complexity of most biological processes is beyond what is comprehensible to the human mind. Therefore, these “systems biology databases” often represent important foundations for quantitative modeling of biological systems. In some cases, these databases allow a direct export of mathematical models. Also, the first collections of mathematical models of biological processes have been developed (databases of models), concerned solely with archiving and curating the models in SBML for future reuse and refinement [131]. Much needed bioinformatics tools for systems biology research are the tools for visualization of network structures and network overlay of simulated and experimental data. These tools include yEd graph editor for editing networks, and tools for visualization of ‘omics data in the context of biochemical networks, such as Cytoscape [132] and Pathway Tools Omics Viewer [133].

## 6. Future Perspectives

Systems biology is rapidly gaining momentum, as evidenced by the number of publications referencing the term (Figure 1). To understand the relevance of “systems” thinking for future biochemical research, one needs only to remember that we know most of the components in many biochemical systems, often in exquisite detail, yet understand very little about how these components interact to produce coherent temporal and spatial behaviors that are the hallmark of biological systems [2]. On the other hand, bioinformatics has originally grown from the need to provide tools and handle increasingly large amounts of biological data. As a discipline bioinformatics continues to grow in this important role, but is also increasingly merging and contributing to systems approaches to provide tools necessary for perhaps the most exciting phase in the development of biological sciences.

One of the defining features of systems biology is the use of mathematical and computational models, which are essential to rigorously account for the inherent complexity of biological systems. This complexity arises from the diversity of components (genes, proteins, and metabolites), the high selectivity of their interactions, and a non-linear nature of these interactions. These properties together render the behavior of biological systems intractable to pure intuition. The computational models used in biochemical systems biology typically require iterative building and stepwise improvements based on the comparison with experiments [134]. Once sufficiently refined, such models have the ability to predict the behavior of the biochemical system under different perturbations, or hypothetical conditions that may be of interest but are not feasible in experimental settings (e.g., when they are too expensive for practical implementation, or when the analysis of many different conditions is desirable [135]). However, in the new era of systems biology, mathematical models are more than just tools for integrating observations, making testable predictions, or for high throughput *in silico* experimentation. Highly refined mathematical models also serve as the embodiments of our current knowledge about specific biochemical systems.

Mathematical and computational models that underpin biochemical studies may involve different levels of detail and scale, depending on the objectives of the study, what is known *a priori*, and what additional information is accessible experimentally. For example, protein complexes may be studied comprehensively [92], or the focus may be on a subset of proteins responsible for a specific function, such as protein import into mitochondria [136]. Most of the so-called bottom-up approaches, which start from the descriptions of interactions, focus on a part of the biological system because we lack a comprehensive information about the system of interest [137]. Nevertheless, bottom-up approaches provide highly useful frameworks for the integration of diverse knowledge, for example, the principles established from decades of biochemical work with the information accessible only with the latest experiments. In contrast, top-down approaches are largely data driven, with the caveat that their comprehensiveness is limited by the limitations in experimental approaches. For example, in one of the most comprehensive metabolomic studies to date, 198 out of an expected 453 primary metabolites were quantified simultaneously in cells grown in minimal medium [138]. Therefore, in such applications advances in technology drive the level of “comprehensiveness” that can be achieved.

Many biochemical processes can be conceptualized as complex dynamic networks on the molecular level (Figure 2), and studies of biochemical networks are assuming centre stage in systems biology [65–67, 139, 140]. Measurements on different ‘omics levels provide different, often complementary views of the functions of molecular networks. Increasingly, we are interested in the crosstalk between the genes, transcripts, proteins, and metabolites that the gene's expression impacts upon [112, 141]. Increasingly sophisticated models will be required to account for increasingly accurate and comprehensive experimental measurements. Systems approaches have already provided a deeper understanding of diverse biochemical processes, from individual metabolic pathways [74, 76], to signaling networks [70–72], to genome-scale metabolic networks [73, 75]. Therefore, we can safely predict that systems thinking will become even more pervasive in future. The role of formal mathematical and computational models in systems approaches renders the role of bioinformatics increasingly important for systems biology research.
